# Knowledge seeking behaviours of pre interns and early career doctors in Sri Lanka: a cross sectional study

**DOI:** 10.1186/s13104-015-1600-3

**Published:** 2015-10-27

**Authors:** Chaturaka Rodrigo, Sachith Maduranga, Milinda Withana, Deepika Fernando, Senaka Rajapakse

**Affiliations:** Department of Clinical Medicine, Faculty of Medicine, University of Colombo, 25, Kynsey Road, Colombo, 00800 Sri Lanka; University Medical Unit, National Hospital, Colombo, Sri Lanka; Department of Parasitology, Faculty of Medicine, University of Colombo, Colombo, Sri Lanka

**Keywords:** Sri Lanka, Medical education, Indexing services, Evidence based practice, Research

## Abstract

**Background:**

Use of reference sources for medical knowledge has changed dramatically over the last two decades with the introduction of online sources of information. This study analyses the medical knowledge seeking behaviours of pre interns and early career doctors in Sri Lanka.

**Methods:**

This cross sectional survey with a convenience sample was conducted at two sites targeting two groups; pre-intern doctors graduated from the Faculty of Medicine, University of Colombo and early career doctors following a postgraduate course at the National Hospital of Sri Lanka. The data collection tool was an online self-administered questionnaire (paper based questionnaires used on request) that probed the patterns of using reference sources for medical knowledge.

**Results and discussion:**

The respondents comprised of 52 pre-interns and 34 early career doctors. A majority (98 %) had internet access. Early career doctors preferred online resources significantly more than the pre-interns. However, the utilization of online resources for evidence synthesis and planning research was unsatisfactory in both groups. A significant proportion (35 %) responded that they had never read a systematic review. Only one person in the entire sample had co-authored a review article.

**Conclusion:**

The use of online resources by participants seems to be satisfactory with a majority shifting to reliable online resources as a reference point for medical knowledge. However, a closer look at the usage patterns reveal that online resources that can be used for more innovative tasks such as evidence synthesis are grossly under-utilized.

## Background

Searching for best evidence and its judicious use in patient care is the core of evidence based practice [1]. It is a skill that has been included into most medical curricula worldwide over the last 20 years [2]. ‘Expert opinion’ driven medical practice of yesteryear has given way to the evidence based practice in the modern era. In the present day, this “evidence”, a massive volume of knowledge that is indexed and stored in virtual space, can be retrieved on demand at the click of a button on a hand-held device from anywhere in the world. Needless to say, trying to remember as much information as possible is a thing of the past. However, with the new technology and explosion of knowledge comes a set of new skills a modern medical practitioner must learn and develop [3]. These include: the ability to filter out quality evidence from unsubstantiated claims or opinions, the ability to critically evaluate available evidence, the ability to read and understand a critical summary on core topics in the form of narrative and systematic reviews, the ability to understand the limitations of evidence and most importantly, skills in proper application of the evidence from a foreign, empirical or a controlled setting individual patients in the local setting [4, 5].

While developed nations have started early on the transition to electronic knowledge hubs, other countries are also following suit [6]. In Sri Lanka, the changes in medical curriculum to prepare the students for online knowledge hubs started as early as 1994 in the countries’ largest medical faculty, the Faculty of Medicine, University of Colombo [5, 7]. The key emphasis in this change was to enable students to become knowledge seekers on their own with guidance by teachers on how to search for knowledge. The role of teachers changed from knowledge givers to knowledge managers who would explain to students how to search for reliable information and how to apply it to a real-life situation. Over the last 20 years, almost all the medical schools in the country have followed this example. In our personal experience as teachers in the Colombo Medical Faculty, fewer students now carry large printed textbooks in the wards. Instead, many students have hand held devices with internet access, and all major textbooks stored within in electronic formats. The traditional “go home and read” has changed to “check online now” or to “google it now”.

While the changes have been made in undergraduate curricula, it is important to determine how the doctors perform after graduating from medical school. Do they slip back to the traditional expert driven medical practice or do they maintain their knowledge seeking behaviour to solve clinical problems? If they do search for information, do they maintain their capacity to search for the best quality evidence according to currently accepted norms?

This study aimed to probe the medical knowledge seeking behaviours of young graduates from the largest medical school of Sri Lanka and early career doctors from the largest health care institution of the country, the National Hospital, Colombo, Sri Lanka.

## Methods

This cross sectional survey with a convenience sample was conducted in two target groups: Pre-interns who are within 1 year since graduation, and early career doctors who are (a) following a postgraduate medical course (either internal medicine or its subspecialties) and (b) within 15 years of clinical practice since graduation. The pre-interns were selected as a separate group as they had just graduated from medical school (and have never practiced). We wanted to see if their knowledge seeking behaviour was different compared to already practicing doctors, as work experience may change a clinician’s perception of useful sources for information.

The study sample consisted of pre-interns who had graduated from the Faculty of Medicine, University of Colombo in 2014 and a sample of early career doctors doing postgraduate studies in the courses specified above from the National Hospital of Sri Lanka. All eligible persons in the above categories were invited to participate (pre-interns were selected as every other person from the roll list). The Faculty of Medicine, Colombo contributes approximately 22 % to the National annual output of 114 medical graduates. However, due to complex nature of transfer procedures, reassignments after examination failures and temporary training attachments, an accurate estimate of the percentage of early career doctors serving in National Hospital of Sri Lanka compared to other teaching hospitals in the country is unavailable. What is known is that this hospital has the highest amount of trainees compared to any other teaching hospital in the country. The data collection tool was an online self-administered questionnaire that was sent via email to participants from each eligible group. Paper versions were printed for those preferring to submit a hardcopy. Due to lack of existing questionnaire applicable to this setting, the researchers developed a questionnaire that consists of 20 items and, on average takes 15–20 min to complete. Apart from two open-ended questions, the rest of the questionnaire consisted of multiple choice questions with options for alternative responses. The questionnaire had sections on knowledge seeking behaviour, sources of online learning, awareness and usage of biomedical databases, indexing services for academic and research purposes. The questionnaire was pre-tested on 10 fourth year medical undergraduates. The data were analyzed using SPSS^®^ statistical software (Version 20, IBM, USA). Findings relevant to descriptive statistics were summarized into proportions and averages (means/SD or percentiles) based on the scales of measurements. Comparisons were evaluated using Chi square test based on retrospective groupings, variable distributional patterns and scales of measurements. Statistical significance was set at p < 0.05. A post hoc power calculation was done for statistically significant differences between the two groups. Ethical clearance for the study was obtained from the ethics review committee of the National Hospital of Sri Lanka.

## Results

The final sample of respondents comprised of 52 pre-interns (males 32 %; mean age ± SD 25.7 ± 0.8 years) and 34 early career doctors (males 77 %; mean age ± SD 33.8 ± 8 years). The response rate for pre-interns and doctors were 54 (52/97) and 72 % (34/47) respectively. Almost the entire sample (98 %) admitted to having regular, on demand access to internet. Of these 87 % had internet access on a hand held mobile device.

Each respondent was asked about their most preferred source of reference for medical knowledge (Table 1). The first choice for a majority (in the entire sample) was a standard printed textbook on the subject (37 %). However, numbers indicating online sources as the first choice was not far behind (30 %). It is notable that almost one-third of the sample (29 %) indicated online sources are their second preferred source of reference. Percentage voting for expert opinion (in house) as the first choice was low (9 %).

**Table 1:** Medical knowledge seeking practices of the sample (n 86)

Variable	Number	Percentage
*Primary source of reference for medical information*		
Textbooks	32	37.2
Online sources (other than textbooks and guidelines)	26	30.2
Guidelines	19	22.1
Expert opinion	8	9.3
Peer opinion	1	1.2
*Most preferred online source for daily medical practice*		
Google searching	19	22.1
Google scholar	8	9.3
Wikipedia	8	9.3
Uptodate	22	25.6
Emedicine	36	41.9
Blogs	3	3.5
Other	1	1.2
*Ever having accessed one of the following indexing services/databases for evidence based knowledge or research*		
PubMed	76	88.4
Cochrane library	34	39.5
Scopus	3	3.5
EMBASE	4	4.7
Web of science	5	5.8
HINARI	67	77.9
*Familiarity with systematic reviews*		
Yes	52	60.5
No	10	11.6
Unclear	24	27.9
*Familiarity with narrative reviews*		
Yes	12	14.0
No	54	62.8
Unclear	20	23.2
*Familiarity with meta*-*analysis*		
Yes	50	58.1
No	3	3.5
Unclear	23	26.7
Correctly identified the difference between a systematic review and a meta-analysis	5	5.8
Unfamiliar with Cochrane reviews	21	24.4
Having ever read a Cochrane systematic review	37	43.0
Active involvement with Cochrane collaboration	1	1.2
Having ever read any review either systematic or narrative	52	60.5
Having authorship in a systematic or a narrative review	1	1.2
*Respondents perception of a reference source for best evidence*		
Guidelines	7	8.1
Journal articles accessed through indexing services	29	33.7
Cochrane reviews	6	7.0
Google scholar	5	5.8
Expert opinion	1	1.2
UpToDate	12	14.0
Medscape/eMedicine	6	7.0
Textbooks	5	5.8
Other/not answered	10	11.6

When asked about the most frequently used online source to obtain general medical information, majority named either Medscape/eMedicine (42 %) [8] or UpToDate (© 2014 UpToDate, Inc., USA) (25.6 %). On academic and research related information, the most popular indexing service accessed was PubMed (88 %) for both groups. The knowledge of other indexing services was relatively poor with less than 40 % of the sample having ever accessed EMBASE, Web of Science, Scopus or Cochrane library collectively. HINARI, a World Health Organization (WHO) initiated portal to access journal archives from low and middle income countries was also very popular with 78 % admitting to frequent use. The usage of non-verified sources such as Wikipedia and blogs were not the primary choice for online referencing.

Of the entire sample, only 60 and 14 % were familiar with the concepts of systematic and narrative reviews respectively. The majority claimed to know about meta-analysis (58 %) but when invited to explain the difference between a systematic review and a meta-analysis, only five respondents (6 %) offered an adequate explanation. Two-thirds of the sample (66 %) chose not to answer this question. Only 43 % of the sample had ever read a Cochrane review. One-fifth (24 %) had never heard of a Cochrane review. Similarly, 35 % had never read a systematic review in any journal.

The differences of responses between the two categories of respondents are summarized in Table 2. The pre-interns were significantly less likely to use online sources as their primary reference source [OR 0.27 (95 % CI 0.1–0.7), p < 0.05, post hoc power: 66 %]. Of the online sources, the pre-interns (although non-significant) preferred Medscape/eMedicine [OR 2.4 (95 % CI 0.96–6.00), p > 0.05] and for early career doctors a significant preference was for UpToDate [OR 0.21 (95 % CI 0.08–0.54), p < 0.05, post hoc power > 80 %]. Interestingly, pre-interns used PubMed for academic search significantly more than the early career doctors [OR infinity, p < 0.05, post hoc power > 80 %]. The use of Cochrane Library was relatively high among both pre interns and early careers doctors, with no significant difference demonstrated between groups. The use of all other indexing services were minimal for both groups.

**Table 2:** Differences in responses among pre interns (n 52) and early career doctors (n 34) with regard to knowledge seeking behaviours

Variable	Pre interns, n (%)	Early career doctors, n (%)	Chi square value	Odd ratio (95 % confidence interval)
Using textbooks as the primary mode of reference	22 (42.3)	10 (29.4)	1.46	1.76 (0.7–4.41)
Using online information as the primary mode of reference	10 (19.2)	16 (47.1)	7.54*	0.27 (0.1–0.7)
Using guidelines as the primary mode of reference	9 (17.3)	10 (29.4)	1.75	0.5 (0.18–1.41)
*Mostly used web based resources*				
Uptodate	12 (23.1)	20 (58.8)	11.24*	0.21 (0.08–0.54)
Emedicine	26 (50)	10 (29.4)	3.58	2.4 (0.96–6.00)
Wikipedia or general google searches	17 (32.7)	9 (26.5)	0.38	1.35 (0.52–3.51)
Having ever used PUBMED for online academic information searching	52 (100)	24 (70.6)	17.31*	NA
Having ever used an alternative indexing service for online academic information searching	20 (38.5)	17 (50)	1.17	0.62 (0.26–1.5)
Having ever used HINARI	38 (73.1)	29 (85.3)	1.78	0.46 (0.15–1.45)
Having ever read a Cochrane systematic review	17 (36.5)	17 (52.9)	2.58	0.49 (0.2–1.18)
Having ever read any systematic review	31 (59.6)	21 (61.8)	0.04	0.61 (0.26–1.45)

* p < 0.01

## Discussion

Use of online resources and e-learning has revolutionized medical education. Having access to a wide variety of databases, images, pictures and videos of educational value at the click of a button have shown to improve outcomes in systematic reviews and meta-analyses of educational research. A systematic review of studies evaluating the benefit of offline (without real time internet connection) e-learning in the fields of medicine, dentistry, nursing and physical therapy showed a benefit in outcomes such as improvements in knowledge, skills and attitudes in participants [9]. None of the studies showed that e-learning was inferior to traditional classroom or textbook based teaching [9]. A systematic review on use of social media based tools for teaching and learning showed better student participation but technical limitations such as availability of online access were noted as challenges [10]. Our sample preferred professional online resources for medical knowledge. However, a number of other studies have shown the educational value of social media tools such as blogs, Youtube, twitter, facebook plus general search engines such as yahoo and google [11]. Still there are some other studies that do not show a marked improvement in dissemination of knowledge by using social media. For example a study conducted in United States showed that the dissemination of clinical practice guidelines of the American Association of Neurology had not significantly increased among physicians and patients even after employing social media, webinars and podcasts [12]. The studies that have assessed the utilization of online resources for continuous medical education are few. A study conducted in 2004 in Lebanon when internet based sources were not as advanced showed that even then of all primary care physicians interviewed (n 178), 98 % used online resources for continuous medical education. It was also shown that participants older than 60 years were significantly more likely to use internet as a source of reference and also to read full text articles compared to younger colleagues [13]. Other studies have also compared online information seeking behaviours between doctors and other health care workers such as nurses. A review into this comparison did not find any significant differences in the knowledge seeking patterns between the two groups [14].

The results our study reveals some interesting observations. Expert opinions no longer guide clinical decision making and was the least important source of reference. The term “expert opinion” in this manuscript refers to on-site opinion of senior physicians. Online sources are in par with textbooks as the primary source of medical education in this era of self-learning. Even though a significant proportion mentioned guidelines as a source of reference, they are also downloaded from online sources except in few situations where local guidelines issued by the Ministry of Health are the primary reference document. It should also be noted that most may have selected to not to report “expert opinion” as the preferred source of knowledge as they are aware that it is not an “accepted” or “politically correct” answer. In the ward setting the decision making might still be guided by “expert opinion”. There are positive aspects of a senior input as well. If the senior doctor actively encourages use of guidelines, reviews and evidence seeking rather than giving the opinion himself, such guidance would be valuable in inculcating an evidence based approach to daily ward rounds. In detail exploration of this is best addressed by a qualitative study and that was not the purpose of this study.

The pattern of online information seeking is also interesting. It is notable that nonspecific google searching and non-verified sources such as Wikipedia were not cited as the most preferred source for online information though many had indicated them as second or third choices. This may be due to the fact that both undergraduate and postgraduate curricula provide strict guidance on referencing from verified professional online resources. The relative preference for UpToDate was statistically significant for early career doctors. Cost may be a direct contributory factor for this difference as eMedicine is available free of charge while UpToDate is a subscription based service. The subscription costs for UpToDate is beyond the reach of many pre-interns who are not still engaged in paid employment. It is also noted that many early career doctors (as opposed to pre-interns), indicated online sources as their main source of medical education. This is not surprising for two reasons. First, in medical school, textbooks are still considered an absolute necessity to get a “proper foundation” in medicine and many reference lists offered by teaching departments still rely heavily on textbooks. Secondly, pre-interns may not be able to afford subscriptions to websites. Therefore, textbooks still remain an integral and a preferred source of information for pre-interns.

It is notable that when it comes to academic and research based information searching PubMed was the most used and preferred option. In fact all pre-interns had used PubMed while nearly 30 % of early career doctors had never used it. The pre-interns were sampled from a single medical school which as a part of its curriculum teaches students on searching for academic and research information on the web and training on using PubMed is mandatory. The doctors in the sample came from different universities and it suggests that the teaching of academic searching in some medical faculties could be improved.

However, apart from Pubmed and Cochrane library, use of other indexing services was extremely low for both categories. Many in both groups had never heard of alternative services such as Scopus, EMBASE and Web of Science. Again this is not surprising as these services are not a priority in medical teaching programmes as they are not free. Only a few institutions such as the National Science Foundation of Sri Lanka have access to Scopus. On the contrary, HINARI, a collaborative effort between major publishers and the WHO which allows access to subscription based journals for citizens of low and middle income countries via a subsidized rate for institutions, was very popular among respondents. Several key institutions such as National Universities and the Post Graduate Institute of Medicine have subscribed for this programme and provide access to its students through their libraries. However, HINARI is an access portal for journal archives. It does not replace the function of an indexing service such as Scopus or EMBASE. The academia and professionals in developing countries are still at a disadvantage in not having access to these services.

The concept of evidence based medicine as understood by respondents also needs scrutiny. The “evidence” for many respondents did not go beyond the recent guidelines. The capacity to read and understand a review article was not satisfactory. In fact, 35 % of the sample had never read a systematic review. When asked about the difference between a narrative and a systematic review, most did not know. Narrative reviews are mainly descriptive and do not follow a specified search criteria. The articles are arbitrarily selected by the authors and hence affected by selection bias. Systematic reviews search the articles in databases in a systematic fashion and have to report inclusion and exclusion criteria according to the PRISMA statement for systematic reviews. Not all systematic reviews are meta-analysis. When there are statistically and methodologically similar studies which have measured similar outcomes the results can be combined in a meta-analysis. Majority were unaware of Cochrane reviews and the quality of evidence it provides. These facts highlight a serious anomaly in undergraduate and postgraduate education in Sri Lanka. This also has an important implication on postgraduate training as the ability to critically evaluate a review and especially a systematic review is essential for a future consultant physician faced with fast changing medical knowledge. Ability to figure out quality of evidence, level of recommendations, summary of evidence and being familiar with the process of systematically evaluating evidence are some of the essential skills to practice as a successful physician and to train juniors in the contemporary knowledge based medical practice. This is especially important when they are functioning as independent consultants in their own units in rural hospitals without any senior supervision.

Though a superficial glance may indicate using the internet for continuous medical education is a positive thing, the use is restricted to certain domains of knowledge and a few “traditional” sources of knowledge (a limited repertoire of websites and online services maintained by professional organizations). Many are not using the vast number of available online resources to critically evaluate and dissect the evidence on a particular question [3, 15]. Only one person in the entire sample had co-authored a review article. It is understandable that a junior doctor may not have the skills or the authority to author a review article by him or herself and they are on most occasions busy with patient care. Still, it is important that senior clinicians extend their support and collaborations to the junior doctors in the process of evidence synthesis. This sample reflects the fact that many junior doctors even in the largest teaching hospital of the country have not had that opportunity. It is clear that much needs to be done in raising awareness of doctors on using online resources to critically analyse and evaluate experimental data, judge and weigh evidence for a particular clinical problem and to plan and execute research to fill the gaps in data. Inculcating the capacity to go beyond the guidelines is important for several reasons: (a) many clinical situations demand a situational judgement as guidelines don’t cover specific issues, (b) many resource limited settings do not have access to treatment strategies advocated by guidelines and (c) proper guidelines do not exist for many neglected tropical diseases that are important for developing countries. In such situations it is the practicing clinicians in these settings that must be in the forefront of summarizing evidence, conducting systematic reviews, identifying gaps in knowledge and designing and executing research to fill the gaps. There are many online services that can help in this process (e.g. Cochrane collaboration). However, this is exactly this critical knowledge and vision that lacks in current undergraduate and postgraduate education in Sri Lanka. While external factors such as cost of subscription based services also play a role, a more important attitude and paradigm shift in medical education needs to be initiated to make the best use of available online medical information services in developing countries such as Sri Lanka.

## Limitations

This sample is from a single medical faculty and a single hospital in Sri Lanka. However both these institutions are the largest institutions in the country in their respective categories. The comparison between pre-interns and doctors does not allow us to interpret that preferences of the former will evolve with time to the preferences of the latter. To establish such an observation, a longitudinal cohort has to be followed up over time.

All early career doctors in this study had enrolled in a postgraduate study programme. The results can be different if a sample was selected from a remote hospital comprising of doctors not engaged in postgraduate studies. However it is unlikely that these statistics will show an improvement if that was the case as learning opportunities and online access are further limited in such settings.

The authors developed the questionnaire for the study as there was no similar questionnaire that served our purpose applicable to this sample. No validity or reliability testing was undertaken with this tool. We conducted a post hoc power analysis for statistically significant differences between the two groups. Out of the three statistically significant differences, one had inadequate (<80 %) power (Table 2).

## Conclusions

This study, assessing the patterns for medical information seeking among pre interns and early career doctors, showed a preference towards textbooks but online sources were also popular especially among early career doctors. The use of online sources for academic and research purposes was less than satisfactory with many responding that they had never accessed some famous online indexing services (less than 50 % of the sample had ever accessed any other service than PUBMED). Again, part of the problem may lie with costs of access but there was a significant minority that had not even made use of freely available resources (through institutional access) such as PubMed and HINARI (24 and 33 % respectively). Using online services to critically analyse clinical questions was also unsatisfactory as there was a significant percentage (35 %) that had never read a systematic review.

## Recommendations

There is a need to improve the knowledge of early career doctors regarding online information searching to plan research and summarize evidence. This is a key area needing urgent attention in a developing nation such as Sri Lanka as it can do a lot with its well-trained health care professionals in contributing to evidence synthesis of neglected tropical diseases and other locally relevant healthcare problems that are not addressed by guidelines and treatment protocols developed elsewhere. Overall, the doctors in developing countries need to shift from a “passive” reading of available information to a more “active” utilization of online resources to assess, analyse and synthesize evidence that are more tailor-made to the local setting.

## References

[1] Glasziou P, Burls A, Gilbert R (2008). Evidence based medicine and the medical curriculum. BMJ.

[2] Coppus S, Emparanza J, Hadley J, Kulier R, Weinbrenner S, Arvanitis T, Burls A, Cabello J, Decsi T, Horvath A (2007). A clinically integrated curriculum in evidence-based medicine for just-in-time learning through on-the-job training: the EU-EBM project. BMC Med Educ.

[3] Mickan S, Tilson JK, Atherton H, Roberts NW (2013). Evidence of effectiveness of health care professionals using handheld computers: a scoping review of systematic reviews. J Med Internet Res.

[4] Cook DA, Levinson AJ, Garside S (2010). Time and learning efficiency in internet-based learning: a systematic review and meta-analysis. Adv Health Sci Edu Theory Prac.

[5] Jayasinghe S (2002). Reforming a conventional medical curriculum.

[6] Leung EY, Malick SM, Khan KS (2013). On-the-job evidence-based medicine training for clinician-scientists of the next generation. Clinical biochem Rev.

[7] Ranasinghe P, Wickramasinghe SA, Wickramasinghe R, Olupeliyawa A, Karunathilaka I (2011). The students’ voice: strengths and weaknesses of an undergraduate medical curriculum in a developing country, a qualitative study. BMC Res Notes.

[8] eMedicine [http://emedicine.medscape.com/]. Accessed 1 May 2014.

[9] Rasmussen K, Belisario JM, Wark PA, Molina JA, Loong SL, Cotic Z, Papachristou N, Riboli-Sasco E, Tudor Car L, Musulanov EM (2014). Offline eLearning for undergraduates in health professions: a systematic review of the impact on knowledge, skills, attitudes and satisfaction. J Glob Health.

[10] Cheston CC, Flickinger TE, Chisolm MS (2013). Social media use in medical education: a systematic review. Acad Med.

[11] Hollinderbaumer A, Hartz T, Uckert F (2013). Education 2.0—how has social media and Web 2.0 been integrated into medical education? A systematical literature review. GMS Zeitschrift fur medizinische Ausbildung.

[12] Narayanaswami P, Gronseth G, Dubinsky R, Penfold-Murray R, Cox J, Bever C, Martins Y, Rheaume C, Shouse D, Getchius TS (2015). The impact of social media on dissemination and implementation of clinical practice guidelines: a longitudinal observational study. J Med Internet Res.

[13] Carney PA, Poor DA, Schifferdecker KE, Gephart DS, Brooks WB, Nierenberg DW (2004). Computer use among community-based primary care physician preceptors. Acad Med.

[14] Younger P (2010). Internet-based information-seeking behaviour amongst doctors and nurses: a short review of the literature. Health Info Libr J.

[15] Brennan N, Mattick K, Ellis T (2011). The Map of Medicine: a review of evidence for its impact on healthcare. Health Info Libr J.

